# The Graphesthesia Paradigm: Drawing Letters on the Body to Investigate the Embodied Nature of Perspective-Taking

**DOI:** 10.1177/2041669517690163

**Published:** 2017-01-01

**Authors:** Gabriel Arnold, Malika Auvray

**Affiliations:** Institut des Systèmes Intelligents et de Robotique, CNRS UMR 7222, Université Pierre et Marie Curie, Paris, France; Département d’Études Cognitives, Institut Jean Nicod (ENS – EHESS – CNRS), École Normale Supérieure, PSL Research University, Paris, France; Institut des Systèmes Intelligents et de Robotique, CNRS UMR 7222, Université Pierre et Marie Curie, Paris, France

**Keywords:** self-consciousness, embodied cognition, spatial perspective-taking, body and self, tactile perception

## Abstract

In this study, we investigated whether adopting a head-centered perspective on the body is an embodied process by means of the graphesthesia task. This task consists of interpreting ambiguous tactile symbols from different spatial perspectives. The results revealed that symbols were more easily recognized when the mental rotation of the head toward the stimulated surface corresponded to physically possible, as opposed to impossible, body movements. Performance also decreased with increasing the amount of body movements that would be necessary to physically rotate the head. These results are in line with an embodied view of spatial perspective-taking, and, more generally, they highlight the important role the body plays in perception.

One key aspect of embodiment consists of emphasizing the role the body plays in shaping the mind. Spatial perspective-taking, that is, mentally displacing the self in a new position and orientation, has been reported to be embodied, as such mental transformation involves not only spatial processes but also motor and somatosensory representations of the body (Kessler & Thomson, 2010). The reliance of bodily self-consciousness on a first-person perspective (Blanke, 2012), together with the view that perspective-taking involves embodied processes, highlights the importance of bodily sensations in the mental manipulations of the self in space.

The graphesthesia task is a promising tool to evaluate the perspectives adopted in perceiving bodily sensations (e.g., Arnold, Spence, & Auvray, 2016). To interpret ambiguous tactile symbols (e.g., the letters b, d, p, and q), people can adopt different perspectives either self-centered (body-centred or head-centred) or other-centered (see Figure 1(a)). The head-centered perspective, which consists of mentally rotating the head toward the stimulated surface, is particularly interesting for investigating embodied perspective-taking as it involves mentally changing the body posture. Crucially, such a head-centered perspective has been described to be constrained by real body movements (Sekiyama, 1991).

**Figure 1.**
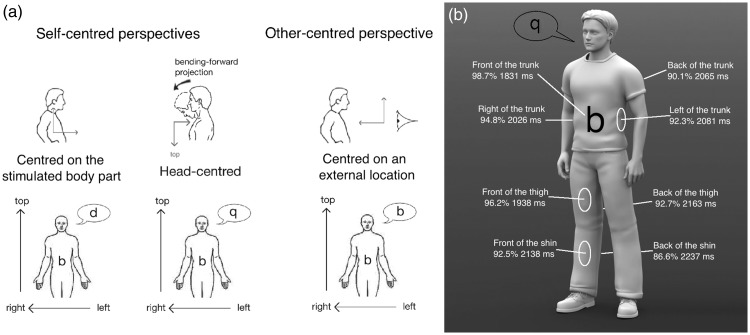
(a) Three possible perspectives and their corresponding responses for the letter “b.” People adopting a head-centered perspective report the 180°-rotated letter “q,” as if the head was bending-forward to see their stomach. (b) Mean accuracy and RTs for the eight stimulated surfaces.

We used the graphesthesia task to investigate whether perspective-taking is influenced by the feasibility and quantity of the movements that would be required to physically see the stimulated body part. We predicted that tactile symbol recognition would be easier for the body surfaces that can be easily viewed (e.g., front) than for those that cannot be viewed (e.g., back), and for the body surfaces that require one body movements (e.g., bending-forward the head to see the belly) than several body movement (e.g., bending-forward the head and the trunk to see the legs). It should be mentioned that two alternatives – not mutually exclusive – would be possible to explain a potential influence of the stimulated body part: mental self-rotation and mental object rotation (see Kessler & Thomson, 2010). However, if mental object rotation was the sole process to be involved, tactile symbol recognition should only depend on the amount of rotation required to align the tactile symbol with the head-centered reference frame, independently of body movements (e.g., the same 180°-rotation for the trunk and the legs).

Thirty-two participants (17 females, 15 males; mean age = 24.8 years, range = 18–40 years) were asked to adopt a head-centered perspective to recognize the letters b, d, p, and q, presented on eight body surfaces (see Figure 1(b)). The letters were presented on the participant’s body by means of a three-by-three matrix of vibrators. The space between vibrators (5 cm) was greater than the tactile spatial resolution of all the stimulated surfaces (see Haggard, Taylor-Clarke, & Kennett, 2003). The letters were traced with sequences of 8 vibrators, 250 ms each, for a total duration of 2,000 ms. The participants completed 8 blocks of 40 trials (10 times each letter) with only one surface stimulated in each block. The order of the blocks was counterbalanced across participants. The participants had to indicate which letter was recognized (four-alternate forced choice), using four adjacent keys on the keyboard. Crucially, as viewing the stimulated body part facilitates tactile perception (Press, Taylor-Clarke, Kennett, & Haggard, 2004), the participants were instructed to not look at the stimulated surface and to keep a standing position with the head oriented forward (see Figure 1(b)).

To investigate the distance from the head, first ANOVAs on accuracy (percentage of correct responses) and response times (RT) were conducted with the orientation of the surface (front and back) and the body part (trunk, thigh, and shin) as within-participant factors, without including the two sides of the trunk. For both accuracy and RTs, there was a significant effect of orientation, accuracy: *F*(1,31) = 7.85, *p* < .01, *η*^2 ^= .202; RT: *F*(1,31) = 17.33, *p* < .001, *η*^2 ^= .359, and body part, accuracy: *F*(2,62) = 6.60, *p* < .01, *η*^2 ^= .175; RT: *F*(2,62) = 8.62, *p* < .001, *η*^2 ^= .217, but no interaction between these factors (*F*s < 1). Performance was better for front (accuracy: 95.8%; RT: 1,969 ms) than for back (89.8%; 2,155 ms) surfaces. The effect of body part was characterized by an effect of the distance from the head: The closer the body part from the head, the better the performance (accuracy: 94.4% for the trunk, 94.4% for the thigh, and 89.5% for the shin; RT: 1,948 ms for the trunk, 2,050 ms for the thigh, and 2,188 ms for the shin). To further investigate the effect of body movements, second ANOVAs were conducted with the surfaces of the trunk only (front, back, left side, and right side) as a within-participant factor. There was a significant effect of surfaces, accuracy: *F*(3,93) = 3.41, *p* < .05, *η*^2 ^= .099; RT: *F*(3,93) = 3.77, *p* < .05, *η*^2 ^= .108. Performance was better for the front (98.7%; 1,831 ms) than for the three other surfaces, accuracy: *F*(1,31) = 10.80, *p* < .01, *η*^2 ^= .258; RT: *F*(1,31) = 17.31, *p* < .001, *η*^2 ^= .358, with no significant differences between these three other surfaces (all *p*s > .108).

Our results show that adopting a head-centered perspective on the body is easier for surfaces that can be easily looked at with real head movements. The head-centered perspective was harder to adopt for surfaces that were either far away from the head (e.g., the shin), physically impossible to be directly looked at (e.g., the back), or necessitating a greater quantity of movements (e.g., the sides compared with the front and the legs compared with the trunk). Our results appear to be more compatible with an embodied perspective-taking than with a mental object-rotation process. Indeed, the angle of rotation necessary to align the tactile stimuli with the head was the same (i.e., 180°) for the belly, the thigh, and the shin. On the other hand, the projection of the head to see the belly requires a bending-forward movement of the head only, whereas for the legs, movements of the head and the trunk are necessary, which explains the effect of the distance from the head. It should be mentioned that the distance effect reported here cannot be explained only by the longer processing time for tactile stimuli further away from the brain. Indeed, the amplitude of the distance effect in our study (around 240 ms/m) is greatly superior to the distance effect reported by Harrar and Harris (2005; 45 ms/m). In addition, this hypothesis would explain only differences in RTs and not the difference in accuracy found in our study.

Overall, these results support the embodied nature of perspective-taking and they extend the results obtained by Kessler and Thomson (2010). Their study showed that mentally taking a spatial perspective on an external scene involves an emulation of the movements that would be necessary to physically place the whole body in a novel position and orientation. The results of our study show that mentally taking a spatial perspective on our own body involves a mental change in body posture that is influenced by physical body parameters. We can thus predict that theactual body posture should affect the process as well. For instance, mentally rotating the head toward the thigh should be easier in a sitting than in a standing position. We can also hypothesize that perspective-taking is affected by other characteristics of the body such as morphology, flexibility, and body size. Such a role of the body is in line with the view that multisensory external information is integrated with information concerning the body state for perception (for a review, see Harris et al., 2015).
